# Draft genome sequence of *Halomonas meridiana* R1t3 isolated from the surface microbiota of the Caribbean Elkhorn coral *Acropora palmata*

**DOI:** 10.1186/s40793-015-0069-y

**Published:** 2015-10-07

**Authors:** Julie L. Meyer, Brian A. Dillard, John M. Rodgers, Kim B. Ritchie, Valerie J. Paul, Max Teplitski

**Affiliations:** Soil and Water Science Department, University of Florida-Institute of Food and Agricultural Sciences, Gainesville, FL USA; Mote Marine Laboratory, Sarasota, FL USA; Smithsonian Marine Station, Fort Pierce, FL USA

**Keywords:** Coral microbiome, Surface mucus layer, Commensal, *Oceanospirillales*, Florida keys

## Abstract

Members of the gammaproteobacterial genus *Halomonas* are common in marine environments. *Halomonas* and other members of the *Oceanospirillales* have recently been identified as prominent members of the surface microbiota of reef-building corals. *Halomonas meridiana* strain R1t3 was isolated from the surface mucus layer of the scleractinian coral *Acropora palmata* in 2005 from the Florida Keys. This strain was chosen for genome sequencing to provide insight into the role of commensal heterotrophic bacteria in the coral holobiont. The draft genome consists of 290 scaffolds, totaling 3.5 Mbp in length and contains 3397 protein-coding genes.

**Electronic supplementary material:**

The online version of this article (doi:10.1186/s40793-015-0069-y) contains supplementary material, which is available to authorized users.

## Introduction

As the name denotes, the first isolated members of the genus *Halomonas* were acquired from saline environments, and members of this halotolerant genus are increasingly isolated from a wide variety of marine environments. While the type species of *Halomonas meridiana* was isolated from an Antarctic saline lake [1], several strains of this species have been isolated from *Acropora* corals, including strain R001 from Palk Bay, India [2] and strains R1t3 and R1t4 from *A. palmata* in the Florida Keys [3]. *Halomonas* spp. have also been identified in surveys of uncultured bacteria in the surface microbiota of *Acropora* corals from the Caribbean and Indonesia [4], while the microbiota of *A. millepora* corals from the Great Barrier Reef are more commonly dominated by members of another genus in the order *Oceanospirillales*, *Endozoicomonas* [5]. Members of the *Oceanospirillales* are increasingly identified as important components of the stable, commensal coral microbiota, and the loss of commensal bacteria is often correlated with disease symptoms [6–8].

Coral-associated commensal bacteria may inhibit pathogens from colonizing the carbon-rich coral mucus layer by outcompeting non-commensals or through the active production of antimicrobial compounds, as previously demonstrated in *Halomonas* strain R1t4 [3]. We chose *H. meridiana* strain R1t3 for whole genome sequencing as a representative coral commensal bacterium from *Acropora* corals. To date, only one other coral commensal bacterial strain has been sequenced: *Endozoicomonas montiporae* from the encrusting pore coral, *Montipora aequituberculata*, isolated from Taiwan [9].

## Organism information

### Classification and features

**Fig. 1:**
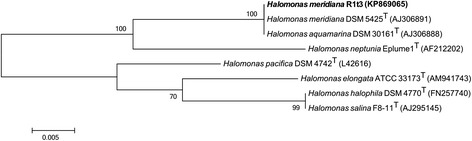
Phylogenetic tree of select *Halomonas* type species and *H. meridiana* strain R1t3. The phylogenetic placement *H. meridiana* strain R1t3 in relation to select type species of marine and salt-tolerant *Halomonas*. Sequences from the 16S rRNA gene were aligned with MUSCLE and trimmed to 1154 bp, the length of the shortest sequence. Evolutionary history was inferred using the Maximum Likelihood method based on the Tamura-Nei model [26]. Branch lengths are measured in the number of substitutions per site. Branch labels indicate the percentage of trees in which the associated taxa were clustered based on 500 bootstraps using MEGA v 5.2.2 [27]. Genome sequences are not currently available for any of the type strains included in this figure

**Fig. 2:**
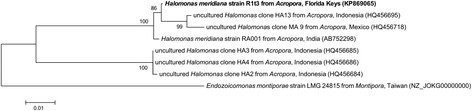
Phylogenetic tree of *H. meridiana* strain R1t3 and other *Halomonas* spp. associated with corals. Sequences from the 16S rRNA gene were aligned with MUSCLE and trimmed to 691 bp, the length of the shortest sequence. Evolutionary history was inferred using the Maximum Likelihood method based on the Tamura-Nei model [26]. Branch lengths are measured in the number of substitutions per site. Branch labels indicate the percentage of trees in which the associated taxa were clustered based on 500 bootstraps using MEGA v 5.2.2 [27]. Genome sequences are currently available for *H. meridiana* strain R1t3 and *Endozoicomonas montiporae* strain LMG 24815

Within the polyphyletic family *Halomonadaceae* [10], *Halomonas* strain R1t3 is a member of the Group 2 assemblage, which may represent a separate genus, however defining characteristics have not been clearly determined for this potential revision [11]. The small subunit ribosomal RNA gene sequence of *Halomonas* strain R1t3 is nearly indistinguishable from the sequence in type strains of both *H. meridiana* and *H. aquamarina* (Fig. 1). Comparison of functional gene loci used in a previously published MLSA study [11] reveal that the loci *secA*, *atpA*, and *rpoD* are approximately 99 % identical between the two type strains and strain R1t3. In contrast, gene sequences for the *gyrB* locus are identical in the type strains, but only 87 % similar to the *gyrB* locus in strain R1t3. Strain R1t3 also exhibits high sequence identity to the small subunit ribosomal RNA gene to strain RA001 isolated from *Acopora* coral in India, and to uncultured *Halomonas* retrieved from *Acropora* corals in Mexico and Indonesia (Fig. 2).

While the strain was originally isolated using sterile coral mucus as a growth substrate [3], subsequent growth in both marine broth and Luria broth have been successful. *H. meridiana* strain R1t3 is aerobic, heterotrophic, and utilizes a wide range of carbon sources, including D-galatonic acid γ-lactone, D-galacturonic acid, D-glucosaminic acid, γ-hydroxybutyric acid, itaconic acid, glycyl-L-glutamic acid, L-phenylalanine, L-serine, L-threonine, phenylethylamine, α-cyclodextrin, Tween 80, N-acetyl-D-glucosamine, D-cellobiose, i-erythritol, α-D-lactose, D-mannitol, putrescine, D,L-α-glycerol phosphate, glucose-1-phosphate, glycogen, Tween 40, and L-asparagine [12]. The carbon sources utilized by the type strains of *H. meridiana* and *H. aquamarina* have been previously documented using Biolog GN2 plates [13] and carbon sources utilized by *Halomonas* strain R1t3 (33E7) have been previously documented using Biolog Ecoplates [12]. Of the 23 substrates in common between the two types of Biolog plates, strain R1t3 can use 12 more substrates than *H. meridiana* and 16 more substrates than *H. aquamarina* (see Additional file 1).

*Halomonas* strain R1t3 grows at 20 to 37 °C in culture, with the highest growth rates at 30 °C (Table 1). No growth was detected at 10 or 50 °C. Strain R1t3 grows at pH 7 to 9, with the highest growth rates at pH 8. Weak growth was detected at pH 6.5 and 10 and no growth occurred at pH 6 and 10.5. Cultures of strain R1t3 produce an unidentified acid during growth, and buffered growth medium at pH 10 was reduced to pH 8 within 24 h of inoculation. Strain R1t3 is halotolerant, exhibiting growth at 2 to 5 % (w/v) sea salt (Coral Pro Salts, Red Sea, Houston, TX) in liquid cultures and growth on 10 % sea salt marine agar. No growth was detected on 20 % sea salt marine agar or at 0 % (w/v) sea salt.

**Table 1:** Classification and general features of *Halomonas meridiana* strain R1t3 [28]

MIGS ID	Property	Term	Evidence code^a^
	Classification	Domain *Bacteria*	TAS [29]
		Phylum *Proteobacteria*	TAS [30]
		Class *Gammaproteobacteria*	TAS [31]
		Order *Oceanospirillales*	TAS [32]
		Family *Halomonadaceae*	TAS [33]
		Genus *Halomonas*	TAS [34]
		Species *Halomonas meridiana*	TAS [1]
		strain: R1t3	
	Gram stain	Negative	NAS
	Cell shape	Rod-shaped	IDA
	Motility	Motile	IDA
	Sporulation	Non-sporulating	NAS
	Temperature range	20–37 °C	IDA
	Optimum temperature	30 °C	IDA
	pH range; Optimum	7–9; 8	IDA
	Carbon source	Varied	TAS [12]
MIGS-6	Habitat	Coral, Marine host	TAS [3]
MIGS-6.3	Salinity	2–10 % NaCl (w/v)	IDA
MIGS-22	Oxygen requirement	Aerobic	TAS [3]
MIGS-15	Biotic relationship	Host-associated	TAS [3]
MIGS-14	Pathogenicity	Non-pathogen	NAS
MIGS-4	Geographic location	Looe Key Reef, Florida	TAS [3]
MIGS-5	Sample collection	April 2005	TAS [3]
MIGS-4.1	Latitude	24° 40′ 48″ N	TAS [3]
MIGS-4.2	Longitude	81° 14′ 24″ W	TAS [3]
MIGS-4.3	Depth	~5 m	TAS [3]
MIGS-4.4	Altitude	Not applicable	

^a^Evidence codes - *IDA* Inferred from Direct Assay, *TAS* Traceable Author Statement (i.e., a direct report exists in the literature), *NAS* Non-traceable Author Statement (i.e., not directly observed for the living, isolated sample, but based on a generally accepted property for the species, or anecdotal evidence). These evidence codes are from the Gene Ontology project [35]

**Fig. 3:**
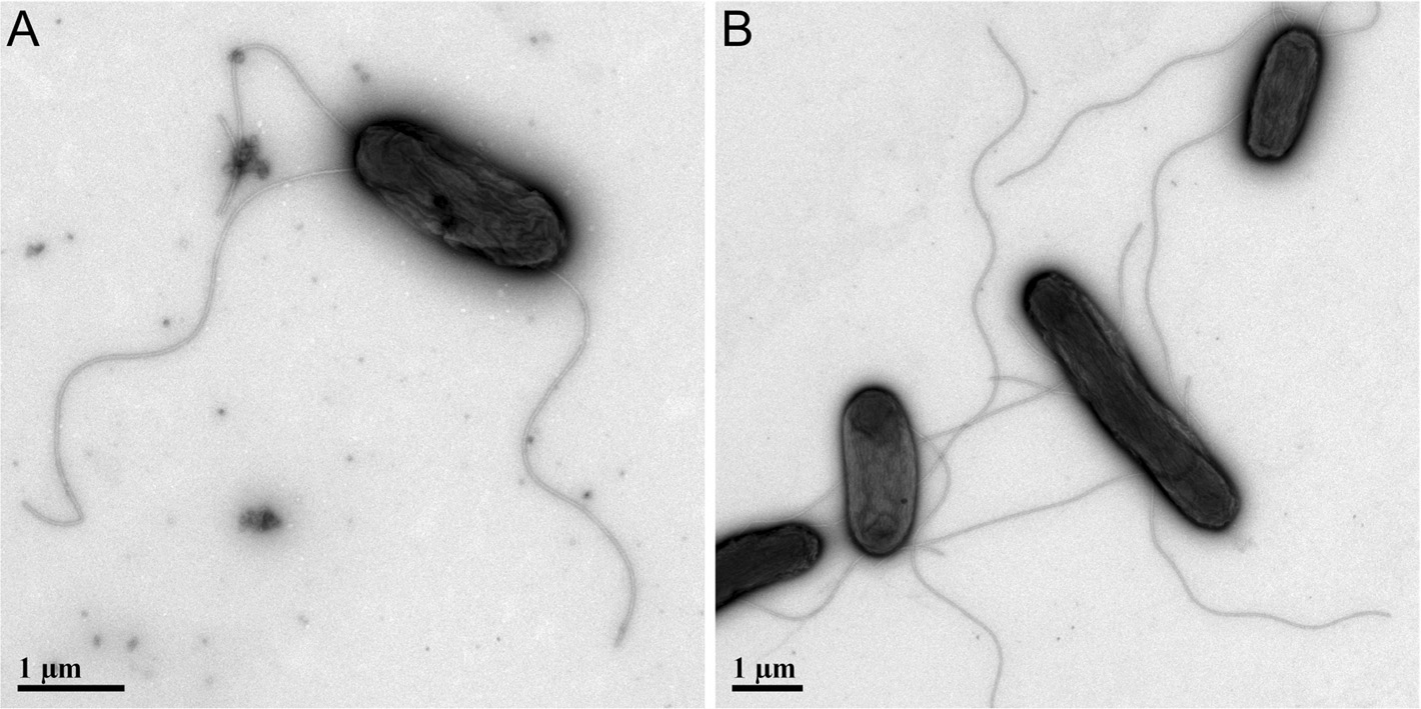
Transmission Electron Micrograph of typical *Halomonas meridiana* strain R1t3 cells. TEM micrograph of strain R1t3 cells grown in marine broth for 18 h and prepared for microscopy with a negative stain. TEM was performed on a Tecnai G2 Spirit 120 kV Transmission Electron Microscope at the University of Florida Electron Microscopy Core. Panel **a** shows a single cell, panel **b** shows multiple cells

Cells of strain R1t3 are around 2 μm long and 1 μm wide (Fig. 3). Cells are motile and multi-flagellated, although the exact number of flagella per cell could not be determined. Colonies grown on marine agar plates are smooth, round, and beige.

#### Symbiotaxonomy

*Halomonas* strain R1t3 was isolated from the surface mucus layer of the scleractinian coral *Acropora palmata* Lamarck 1816 (commonly known as Elkhorn Coral), from the Florida Keys National Marine Sanctuary (Table 1). *A. palmata* historically dominated shallow Caribbean reefs, but is currently listed as Critically Endangered on the IUCN Red List of Threatened Species due to extensive losses from white-band disease, climate change, and human-related impacts [14].

## Genome sequencing information

### Genome project history

*H. meridiana* strain R1t3 was chosen for genome sequencing as a representative of the stable, commensal bacterial community inhabiting the dynamic surface mucus layer of an acroporid coral. The genome project information is available through the Genomes On Line Database [15] and the annotated genome sequences are publicly available through both the Integrated Microbial Genomes (IMG) portal [16] and GenBank (Table 2).

**Table 2:** Genome sequencing project information

MIGS ID	Property	Term
MIGS 31	Finishing quality	Draft
MIGS-28	Libraries used	Illumina DNA-seq, PE library (~350 bp insert size)
MIGS 29	Sequencing platforms	Illumina HiSeq
MIGS 31.2	Fold coverage	23X
MIGS 30	Assemblers	IDBA-UD 1.1.0
MIGS 32	Gene calling method	IMG: DOE-JGI Genome Annotation Pipeline, NCBI Prokaryotic Genome Annotation Pipeline
	Locus Tag	IMG: Halo, NCBI: VE30
	Genbank ID	JZEM00000000
	GenBank Date of Release	04 March 2015
	GOLD ID	Gp0103707
	BIOPROJECT	PRJNA269585
MIGS 13	Source Material Identifier	NCMA B79
	Project relevance	Host-associated

### Growth conditions and genomic DNA preparation

A culture of *Halomonas meridiana* R1t3 (National Center for Marine Algae & Microbiota, Bigelow Laboratory for Ocean Sciences, Accession # NCMA B79) was grown from a single colony at room temperature in 5 ml of Difco™ Marine Broth 2216 for 48 h. Cells were separated from the culture medium using microcentrifugation (12,000 rpm for 5 min) and genomic DNA (gDNA) was extracted from the pelleted cells with a Qiagen AllPrep DNA/RNA Micro Kit (Germantown, MD). The quality of the extracted gDNA was assessed by visualization on a 1 % agarose gel stained with ethidium bromide and with a BioAnalyzer DNA chip, then sent to the University of Maryland Institute for Bioscience and Biotechnology Research for library preparation and sequencing.

### Genome sequencing and assembly

A genomic library was prepared with a TruSeq DNA Sample Preparation Kit (Illumina, San Diego, CA) and sequenced on an Illumina HiSeq with the high-output, 100-bp paired-end protocol at the University of Maryland Institute for Bioscience and Biotechnology Research. The average insert size was 337 bp with a DNA concentration of 192 nM. Sequencing reads were quality-filtered by trimming adaptors with cutadapt [17] and filtering reads for a minimum quality score of 30, minimum length of 100 bp, and discarding all sequences with ambiguous base calls using Sickle [18]. The unassembled, quality-filtered reads (41,481,885 read pairs) are publicly available through the NCBI Sequence Read Archive (SRA) under the accession number SRX800904. Quality-filtered reads were interlaced with the shuffleSequences_fastq.pl script from velvet [19] and assembled with IDBA-UD [20] with k-mer sizes of 60, 70, and 80. This assembly yielded 290 contigs greater than 150 bp, a maximum contig length of 173,110 bp, and a total assembly length of 3.5 Mbp. The estimated Illumina sequencing coverage is 23×. To evaluate the quality of the assembly, unassembled reads were mapped to the 290 assembled contigs with bowtie2 [21] and alignment statistics were recovered with samtools [22]. The overall alignment rate was 99.9 %. The coverage of the genome was further assessed from the unassembled reads using nonpareil [23], which gave an estimated coverage of 100 %, indicating the sequencing effort was more than sufficient to capture all of the genome (4.2 Gbp actual effort, compared to 150 Mbp estimated required effort). Whole genome alignment of the draft genome sequences of *Halomonas* strain R1t3 and *Endozoicomonas montiporae* strain LMG 24815 was performed with Mauve v2.4 [24].

### Genome annotation

The draft genome assembly was submitted to IMG-ER [16] for annotation (Taxon ID 2588254266, publicly available) and discussion of genome content here is restricted to the IMG annotation. The 130 contigs greater than 500 bp were also submitted to GenBank (JZEM00000000) and annotated through the NCBI Prokaryotic Genome Annotation Pipeline. Locus tags in IMG are prefaced by “Halo” while locus tags in GenBank are prefaced by “VE30”.

## Genome properties

The draft genome of strain R1t3 is comprised of 290 scaffolds, with a total length of 3.5 Mbp (Table 3). Compared to the other 28 genomes of *Halomonas* currently in the IMG database (as of April 2015), which range from 2.8 Mbp to 5.9 Mbp, the genome of strain R1t3 is smaller than the average *Halomonas* genome size of 4.3 Mbp. The G + C content is 57 %, while the other *Halomonas* genomes contain 52 to 68 % G + C. A total of 3526 genes were annotated through the IMG pipeline, with approximately 70 % genes assigned to Clusters of Orthologous Genes (Table 4). No pseudogenes or CRISPR repeats were detected.

**Table 3:** Genome statistics based on the IMG Annotation Pipeline

Attribute	Value	% of Total
Genome size (bp)	3,507,875	100.00
DNA coding (bp)	3,136,266	89.41
DNA G + C (bp)	1,986,943	56.64
DNA scaffolds	290	100.00
Total genes	3526	100.00
Protein coding genes	3397	96.34
RNA genes	129	3.66
Pseudo genes	0	0.00
Genes in internal clusters	2549	72.29
Genes with function prediction	2887	81.88
Genes assigned to COGs	2469	70.02
Genes with Pfam domains	2945	83.52
Genes with signal peptides	259	7.35
Genes with transmembrane helices	811	23.00
CRISPR repeats	0	0

**Table 4:** Number of genes associated with general COG functional categories, based on the IMG Annotation Pipeline

Code	Value	% age	Description
J	207	7.53	Translation, ribosomal structure and biogenesis
A	1	0.04	RNA processing and modification
K	179	6.51	Transcription
L	115	4.18	Replication, recombination and repair
B	2	0.07	Chromatin structure and dynamics
D	32	1.16	Cell cycle control, Cell division, chromosome partitioning
V	63	2.29	Defense mechanisms
T	132	4.80	Signal transduction mechanisms
M	171	6.22	Cell wall/membrane biogenesis
N	71	2.58	Cell motility
U	36	1.31	Intracellular trafficking and secretion
O	128	4.65	Posttranslational modification, protein turnover, chaperones
C	195	7.09	Energy production and conversion
G	149	5.42	Carbohydrate transport and metabolism
E	253	9.20	Amino acid transport and metabolism
F	72	2.62	Nucleotide transport and metabolism
H	155	5.64	Coenzyme transport and metabolism
I	136	4.95	Lipid transport and metabolism
P	176	6.40	Inorganic ion transport and metabolism
Q	64	2.33	Secondary metabolites biosynthesis, transport and catabolism
R	202	7.35	General function prediction only
S	147	5.35	Function unknown
-	1057	29.98	Not in COGs

The total is based on the total number of protein coding genes in the genome

## Insights from the genome sequence

Like other members of the *Halomonadaceae*, strain R1t3 exhibits tolerance to a wide range of salinities that is likely mediated through the production of osmoprotectants, such as glycine betaine. Strain R1t3 has homologues of the two genes needed to produce glycine betaine. These genes, choline dehydrogenase (Halo_00078/VE30_01315) and betaine aldehyde dehydrogenase (Halo_00077/VE30_01310) are part of a operon and are preceded by a choline ABC transporter periplasmic binding protein (Halo_00075/VE30_01300) and a TetR-family transcriptional regulator (Halo_00076/VE30_01305). The genome of strain R1t3 also contains a biosynthetic cluster (*ectABC*) for the production of the cyclic amino acid osmolyte, ectoine (Halo_01324/01325/01326, VE30_07080/07085/07090) as well as ectoine utilization genes *eutED* (Halo_01398/01399, VE30_04620/04625).

The genome of strain R1t3 reflects its ability to utilize a wide range of carbon sources, including gene homologues for six different glycoside hydrolases (GH), used for breaking down complex carbohydrates. Four belong to GH family 13 (Halo_01730/VE30_08480, Halo_01736/VE30_08510, Halo_02655/VE30_13740, Halo_02891/VE30_RS10055), used for the breakdown of starch and glycogen. Single genes encode for GH family 3 (Halo_00185/VE30_02710) and GH32 (Halo_01720/VE30_08435) glycosidases, which act on oligosaccharides and fructan, respectively. The genome of strain R1t3 also contains homologues of genes required for glycerol transport across the membrane (*glpSTPQV*) (Halo_00080/00081/00082/00083/00085. VE30_01325/01330/01335/01340/01350) and glycerol degradation (*glpAD*) (Halo_00086/VE30_01355). The efficient use of multiple sources of carbon may be mediated through the widely conserved *csrA* carbon storage regulator (Halo_02194/VE30_11745) that is present in the genome.

Previous work examining the utilization of coral mucus as a carbon source in this strain demonstrated that glucose and galactose are preferred carbon sources for strain R1t3 [25]. The addition of glucose to media containing high-molecular-weight components of coral mucus repressed the enzymatic activity of α-D-fucopyranosidase and the addition of galactose repressed α-L-galactopyranosidase activity. This catabolite repression is likely effected through the *tctE/D* two-component system (Halo_03014/VE30_14870, Halo_03015/VE30_14875) and *tctCBA* tricarboxylate transport membrane protein (Halo_03016/03017/03018, VE30_14880/14885/14890) encoded in the genome.

Overall, the average nucleotide identity (ANI) between the IMG annotated draft genomes of *H. meridiana* strain R1t3 (3.5 Mbp) and *Endozoicomonas montiporae* LMG 24815 (5.6 Mbp) was 68.64 %. Orthologs shared between the two genomes were identified using a minimum of 60 % sequence identity and 70 % coverage. Despite the similarity of the ecological niches filled by these two *Oceanospirillales* bacteria, only 11 % of the genes in *Halomonas* strain R1t3 (392 genes) have orthologs in the *Endozoicomonas* genome. Reducing the threshold to 30 % sequence similarity only increased the total proportion of orthologs to roughly 12.5 % (442 genes). Of the orthologs with at least 30 % sequence identity, three of the four starch/glycogen-degrading glycoside hydrolases and the single oligosaccharide-degrading GH in *Halomonas* had orthologs in *Endozoicomonas*.

## Conclusions

The draft genome sequence of *Halomonas meridiana* strain R1t3 provides insight for the role of a representative strain of the commensal bacterial community associated with the surface mucus layer of an *Acropora* coral. Strain R1t3 can utilize a wide range of carbon sources, as demonstrated in culture and supported by genome content.

## Additional file

Additional file 1: Table S1. Utilization of carbon sources by *Halomonas* species. Description of data: Comparison of the utilization of carbon sources between *Halomonas meridiana* R1t3 and the type strains of *H. meridiana* and *H. aquamarina*. (PDF 85 kb)
