# Supplementary material for: Draft genome sequence of Halomonas meridiana R1t3 isolated from the surface microbiota of the Caribbean Elkhorn coral Acropora palmata
Source: Stand Genomic Sci. 2015 Oct 7;10:75. doi: 10.1186/s40793-015-0069-y (PMC4597393; doi:10.1186/s40793-015-0069-y)
Supplement: Additional file 1: Table S1. — Utilization of carbon sources by Halomonas species. Description of data: Comparison of the utilization of carbon sources between Halomonas meridiana R1t3 and the type strains of H. meridiana and H. aquamarina. (PDF 85 kb) [file 40793_2015_69_MOESM1_ESM.pdf]

Supplemental Table 1. Utilization of carbon sources by *Halomonas* species[a].

**Carbon Source**	***H. meridiana*** **R1t3**	***H. meridiana*** **DSM 5425^T**	***H. aquamarina*** **DSM 30161^T**
D-galactonic acid γ-lactone	+	-	-
D-galacturonic acid	+	-	-
D-glucosaminic acid	+	+	+
γ-hydroxybutyric acid	+	-	-
Itaconic acid	+	-	-
Glycyl-L-glutamic acid	+	-	-
L-phenylalanine	+	+	-
L-serine	+	+	-
L-threonine	+	+	-
Phenylethylamine	+	-	-
α-cyclodextrin	+	+	+
Tween 80	+	-	+
N-acetyl-D-glucosamine	+	-	-
D-cellobiose	+	-	-
i-erythritol	+	-	-
α-D-lactose	+	+	+
D-mannitol	+	+	-
Putrescine	+	-	+
D,L-α-glycerol phosphate	+	-	-
Glucose-1-phosphate	+	+	_
Glycogen	+	+	+
Tween 40	+	+	+
L-asparagine	+	+	-

[a]Carbon sources used by the type strains of *H. meridiana* and *H. aquamarina* were tested with a Biolog GN2 plate (5) and carbon sources used by strain R1t3 were tested with a Biolog Ecoplate (6).
